# Lower seroprevalence of *Toxoplasma gondii* in swine from central China after an outbreak of African swine fever

**DOI:** 10.1051/parasite/2021053

**Published:** 2021-07-02

**Authors:** Weitao Xie, Shilin Xin, Nan Jiang, Gaiping Zhang, Longxian Zhang, Xiangrui Li, Yurong Yang

**Affiliations:** 1 MOE Joint International Research Laboratory of Animal Health and Food Safety, College of Veterinary Medicine, Nanjing Agricultural University 210095 Nanjing PR China; 2 College of Veterinary Medicine, Henan Agricultural University 450046 Zhengzhou PR China

**Keywords:** *Toxoplasma gondii*, Seroepidemiology, Swine, African swine fever, China, Risk factors

## Abstract

*Toxoplasma gondii* is widespread worldwide and can infect swine. This study evaluated the seroprevalence of *T. gondii* in swine from central China after an outbreak of African swine fever (ASF). A total of 2683 swine serum samples were collected from farms in four provinces. Of the serum samples, 1.42% (38/2683) (95% CI, 1.03–1.94) tested positive for *T. gondii* IgG antibody by a modified agglutination test (MAT) (cut-off: 1:25). Comparing with the results of previous studies, specifically our survey from before the outbreak, the seroprevalence of *T. gondii* in swine from central China was significantly decreased after the occurrence of ASF (OR = 7.679, 2015–2017 vs. 2019–2020). In general, the proportion of seropositive animals increased with the age of the swine, indicating post-natal transmission of *T. gondii.* Furthermore, there was a significant difference in seroprevalence between suckling pigs and weaned pigs (*p* < 0.05). This is the first large-scale investigation of *T. gondii* infection in swine after an ASF outbreak in China. The lower seroprevalence of *T. gondii* in swine after ASF may be due to stricter biosecurity measures on the farms, but results indicated swine exposure to zoonotic parasites despite these measures. This highlights that pigs must be considered a potential source of human *T. gondii* infections.

## Abstract (fr)

*Toxoplasma gondii* est répandu dans le monde entier et peut infecter les porcs. Cette étude a évalué la séroprévalence de *T. gondii* chez les porcs du centre de la Chine après une épidémie de peste porcine africaine (PPA). Au total, 2 683 échantillons de sérum de porc ont été prélevés dans des fermes de quatre provinces. Parmi les échantillons de sérum, 1,42 % (38/2683) (IC à 95 %, 1,03–1,94) étaient positifs pour les anticorps IgG de *T. gondii* par un test d’agglutination modifié (MAT) (seuil : 1:25). En comparaison avec les résultats d’études précédentes, en particulier notre enquête d’avant l’épidémie, la séroprévalence de *T. gondii* chez les porcs du centre de la Chine a été significativement diminuée après l’apparition de la PPA (OR = 7,679, 2015-2017 vs 2019-2020). En général, la proportion d’animaux séropositifs augmentait avec l’âge des porcs, indiquant une transmission postnatale de *T. gondii*. De plus, il y avait une différence significative entre la séroprévalence chez les cochons de lait et les cochons sevrés (*p* < 0,05). Il s’agit de la première enquête à grande échelle sur l’infection à *T. gondii* chez le porc après l’épidémie de PPA en Chine. La séroprévalence plus faible de *T. gondii* chez les porcs après la PPA peut être due à des mesures de biosécurité plus strictes dans les fermes, mais les résultats ont indiqué une exposition des porcs aux parasites zoonotiques malgré ces mesures. Cela souligne que les porcs doivent être constamment considérés comme une source potentielle d’infection humaine à *T. gondii*.

## Introduction

*Toxoplasma gondii* is a widely distributed obligate intracellular parasitic protozoan that infects virtually all warm-blooded animals, including humans and swine [5]. About 1800 million people worldwide are chronically infected with *T. gondii* [14, 17]. Infection with *T. gondii* is usually asymptomatic in healthy people, but can be severe in people with immunodeficiency [5]. There are two main modes of transmission of *T. gondii*: horizontal transmission, mainly through oral infection, and vertical transmission through the placenta [11, 15].

Swine are susceptible to *T. gondii*, with seroprevalence ranging from 0% to 96.6% in different regions of the world [8]. China is the largest pork producer and consumer worldwide [18]. The positive serum rate of *T. gondii* in China was 32.9% from 2000 to 2017 [4], indicating that *T. gondii* infection is widespread on pig farms, which resulted in an adverse impact on the farmer’s income and human health. On August 3, 2018, there was an African swine fever (ASF) outbreak in China. Measures (early virus detection by clinical signs and laboratory diagnosis, and strict biosecurity) were implemented to control the spread of this disease [1–3]. This study investigated the seroprevalence of *T. gondii* infection in swine from Chinese farms and compared the seroprevalence before and after the ASF outbreak.

## Materials and methods

### Ethics approval and consent to participate

This study was conducted following the guideline recommendations for using samples from animals by the Beijing Association for Science and Technology (SYXK [Beijing] 2007-0023). The swine sera were collected with agreement from the farmers. The ethics committee of the Henan Agricultural University (China) further approved this study.

### Investigation sites and serum samples

**Figure 1:**
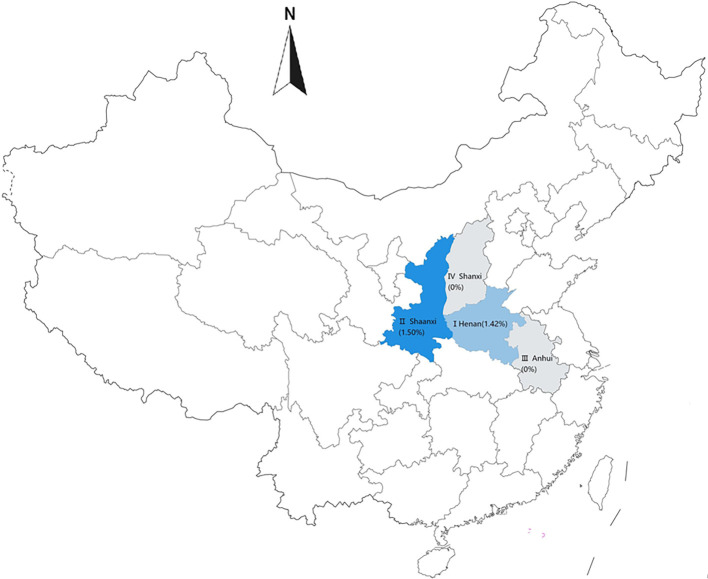
Seroprevalence of *T. gondii* in swine from central China. I: Henan; II: Shaanxi; III: Anhui; IV: Shanxi. Map adapted from http://bzdt.ch.mnr.gov.cn/.

In this study, 2683 swine serum samples were collected from 16 farms in Henan (*n* = 5), Shaanxi (*n* = 9), Anhui (*n* = 1), and Shanxi (*n* = 1) provinces in 2019–2020 (Table 1 and Fig. 1). These blood samples were collected from live animals by veterinarians on the farms. The sera were used for porcine reproductive and respiratory syndrome and porcine circovirus antibodies screening, which also allowed us to survey *T. gondii* infection. These serum samples were transported to the Henan Agricultural University (Zhengzhou, Henan, China) in cooler boxes. Information including collection dates, age, sex, health (no abnormal clinical signs), unhealth (anorexia, fever, cough, miscarriage and diarrhea), whether pregnant, and farm locations (Henan, Shaanxi, Anhui and Shanxi) was also collected for the samples.

**Table 1:** Seroepidemiology and background information on *Toxoplasma gondii* in 2683 swine.

	Province	No. of samples	MAT titers								% (positive no.)	95% CI
			1:25	1:50	1:100	1:200	1:400	1:800	1:1600	1:3200		
Location	Henan	703	7	2	0	0	1	0	0	0	1.42% (10)	0.74–2.64
	Shaanxi	1867	10	13	3	1	1	0	0	0	1.50% (28)	1.03–2.17
	Anhui	27	0	0	0	0	0	0	0	0	–	0–14.76
	Shanxi	86	0	0	0	0	0	0	0	0	–	0–5.13
	Total	2683	17	15	3	1	2	0	0	0	1.42% (38)	1.03–1.94
Sampling time	2019	265	5	2	0	0	0	0	0	0	2.64% (7)	1.18–5.46
	2020	2418	12	13	3	1	2	0	0	0	1.28 (31)	0.90–1.82
	Total	2683	17	15	3	1	2	0	0	0	1.42% (38)	1.03–1.94
Growth stages	Suckling pig	213	0	0	0	0	0	0	0	0	–	0–2.13
	Nursery pig	452	2	0	0	0	0	0	0	0	0.44% (2)	0.01–1.71
	Fattening pig	595	10	1	2	0	0	0	0	0	2.18% (13)	1.24–3.74
	Adult pig	1328	5	14	1	1	2	0	0	0	1.73% (23)	1.14–2.60
	Total	2588	17	15	3	1	2	0	0	0	1.47% (38)	1.07–2.01
Sex	Male	104	0	1	1	0	0	0	0	0	1.92% (2)	0.10–7.17
	Female	1276	7	11	0	1	1	0	0	0	1.57% (20)	1.00–2.42
	Total	1380	7	12	1	1	1	0	0	0	1.59% (22)	1.04–2.42
Pregnancy	Yes	498	2	5	0	1	0	0	0	0	1.61% (8)	0.76–3.19
	No	292	4	6	0	0	0	0	0	0	3.42% (10)	1.79–6.27
	Total	790	6	11	0	1	0	0	0	0	2.28% (18)	1.42–3.60
Health condition	Health	1821	13	15	3	1	1	0	0	0	1.81% (33)	1.29–2.54
	Unhealthy	130	0	0	0	0	0	0	0	0	0%	0–3.45
	Total	1951	13	15	3	1	1	0	0	0	1.69% (33)	1.20–2.37
Parity	Multiparous	576	3	6	0	0	1	0	0	0	1.74% (10)	0.90–3.21
	Primiparous	104	2	0	0	0	0	0	0	0	1.92% (2)	0.10–7.17
	Total	680	5	6	0	0	1	0	0	0	1.76% (12)	0.98–3.09

### Serological testing

All the serum samples were tested for antibodies against *T. gondii* by a modified agglutination test (MAT) [6]. Sera with MAT titers of 1:25 or higher were considered positive for *T. gondii* [5]. Whole formalin-treated *T. gondii* tachyzoites were obtained from the University of Tennessee Research Foundation (Knoxville, TN, USA; https://utrf.tennessee.edu/). *Toxoplasma gondii*-positive mouse sera were provided by Dr. J. P. Dubey (ARS, USDA, Beltsville, MD, USA) as reference sera. All the serum samples were tested at 1:25, after which the dilution was doubled to 1:3200, and negative and positive controls were included in each plate.

### Statistical analysis

The pig population was divided into four stages: suckling piglets (0–4 weeks), nursery pigs (>4 weeks to 10 weeks), fattening pigs (>10 weeks to 20 weeks), and adult pigs (>20 weeks). In addition, the swine farm locations, pregnancy, number of deliveries, and sampling time were also compared. The classification standards are summarized in Table 2.

**Table 2:** Seroprevalence and risk factors for *Toxoplasma gondii* in swine tested by modified agglutination test.

Factor	Classification standards	Classification standards	No. of samples	Seroprevalence (%)	Odds ratio (95% confidence interval)	*p*-value
Province	Shaanxi	–	1867	1.50%	1.005 (0.5098–2.184)	0.8850
	Henan	–	703	1.42%	1	
Growth stages	Fattening pig	11–20 week	595	2.18%	9.896 (0.5853–167.3)	0.0296^a^,*
	Adult pig	>21 week	1328	1.73%	5.026 (1.128–22.39)	0.0188^b^,*
	Nursery pig	5–10 week	452	0.44%	3.966 (0.9309–16.89)	0.0442^c^,*
	Suckling pig	0–4 week	213	0%	1	
Sex	Male	–	104	1.92%	1.231 (0.2837–5.344)	0.7807
	Female	–	1276	1.57%	1	
Pregnancy	No	Replacement gilt, waiting for breeding	292	3.42%	2.172 (0.8473–5.568)	0.0983
	Yes	–	498	1.61%	1	
Health condition	Health	–	1821	1.81%	4.889 (0.2977–80.29)	0.1216
	Unhealthy	Illness, loss of appetite, miscarriage	130	0%	1	
Parity	Primiparous	–	104	1.92%	1.110 (0.2396–5.141)	0.8940
	Multiparous	–	576	1.74%	1	
Sampling time	2015–2017	–	2798^d^	9.94%	7.679 (5.447–10.83)	<0.0001*
	2019–2020	This study	2683	1.42%	1	

Note: *Statistically significant (*p* < 0.05).

a Fattening pigs versus suckling pigs.

b Fattening pigs versus nursery pigs.

c Nursery pigs versus adult pigs.

d Data were obtained from Su et al. [16].

These samples were collected from central China and detected by MAT.

Statistical analysis was performed using GraphPad Prism 8.0 software (GraphPad Software Inc., San Diego, CA, USA). The results were analyzed by the Chi-square or Fisher’s exact test and the Monte Carlo test of simulated data to assess the risk factors associated with *T. gondii* infection. A *p* value of <0.05 was considered statistically significant.

## Results

The total seroprevalence of *T. gondii* antibodies was 1.42% (38/2683, 95% CI, 1.03–1.94) in swine. Titers of 1:25 in 17, 1:50 in 15, 1:100 in three, 1:200 in one, and 1:400 in two were found (Table 1). The seroprevalence values for *T. gondii* in swine were 2.64% (7/265, 95% CI, 1.18–5.46) in 2019 and 1.28% (31/2418, 95% CI, 0.90–1.82) in 2020, but there was no significant difference between them (*p* = 0.0754). In Henan and Shaanxi, the seroprevalence values of *T. gondii* were 1.42% (10/703, 95% CI, 0.74–2.64) and 1.50% (28/1867, 95% CI, 1.03–2.17), respectively. However, the difference between the two provinces was not statistically significant (*p* = 0.8850). Also, no positive serum in swine was observed in Anhui (0/27) and Shanxi (0/86) provinces (Tables 1 and 2, Fig. 1).

The swine were divided into four groups according to their growth stages. No *T. gondii* antibodies were detected in suckling pigs (0/213). The seroprevalence of *T. gondii* was 0.44% (2/452, 95% CI, 0.01–1.71) for nursery pigs, 2.18% (13/595, 95% CI, 1.24–3.74) for fattening pigs, and 1.73% (23/1328, 95% CI, 1.14–2.60) for adult pigs. A comparison of different growth stages showed that the seroprevalence of *T. gondii* in pigs at the fattening stage was significantly higher than in suckling pigs (*p* = 0.0296) and nursery pigs (*p* = 0.0188). In addition, the seroprevalence of *T. gondii* in adult pigs was significantly higher than that in nursery pigs (*p* < 0.05), and there was no significant difference in other stages. In terms of males and females, pregnant sows and non-pregnant sows, primiparous sows and multiparous sows, there were no significant differences (*p* > 0.05) (Tables 1 and 2).

## Discussion

**Figure 2:**
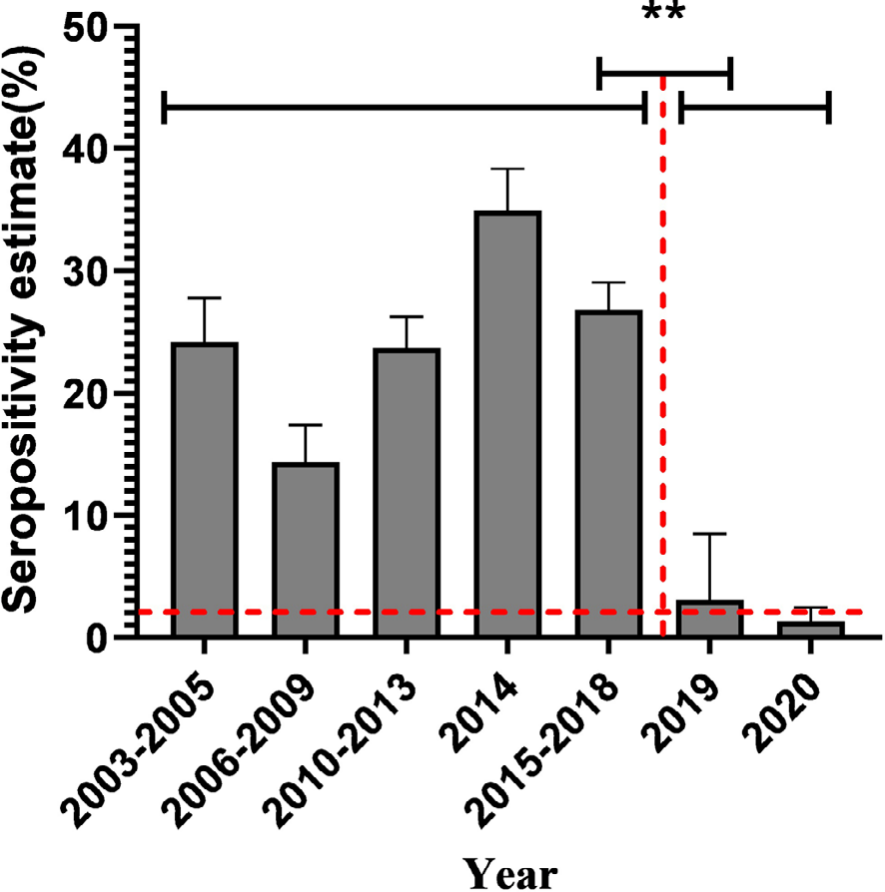
Comparison of seroprevalence of *T. gondii* in swine from four provinces before and after 2018. The four provinces were Henan, Shaanxi, Anhui and Shanxi. Data were obtained from Dong et al. [4] and Dubey et al. [8]. **p < 0.001.

Pork production is an important economic industry for many countries, including China. *Toxoplasma gondii* infection in swine threatens pork production and food security [8]. Therefore, it is necessary to understand the *T. gondii* infection status in swine from China. In this survey (2019–2020), the seroprevalence of *T. gondii* detected by MAT was 1.42% (38/2683) in swine from central China. According to the isolation results of *T. gondii* from 1000 swine hearts by bioassays in mice or cats, the percentage of isolation of viable *T. gondii* from cardiac tissue with MAT antibody titers in swine was: 37.1% (1:20), 38.1% (1:40), 60% (1:80), 75% (1:200) [7]. A titer of 1:25 in 17, 1:50 in 15, 1:100 in three, 1:200 in one, and 1:400 in two were found in this study (Table 1), which indicated that about 15 swines contained viable *T. gondii* parasites. The seroprevalence survey of *T. gondii* in this study (2019–2020) was lower than the results for swine sampled from 2015 to 2017 (9.94%, 278/2798) (*p* < 0.05); these samples were also collected from central China and tested using MAT [16]. The seroprevalence of *T. gondii* in swine from four provinces (Henan, Shaanxi, Anhui, and Shanxi) from 2003 to 2018 was summarized, and it was found to be higher than the seroprevalence obtained in this study (*p* < 0.05) (Fig. 2). Thus, the seroprevalence of *T. gondii* in swine significantly decreased after 2018. This difference may be related to the outbreak of ASF in China. The African swine fever virus (ASFV) first appeared in China in 2018 and spread rapidly to several provinces. ASFV was transmitted fast and showed a high mortality rate [3]. ASFV could be transmitted through various ways, including pigs and pork movement, the water and food supply chain, birds, arthropods, mammals, and mechanical vectors [3, 19]. At the beginning of the ASF outbreak, strict biosecurity measures were implemented to control this disease on all swine farms in China. Treatment measures included restricting human access to the farm, control of rats, mice, cats, birds, flies, and mosquitoes, frequent cleaning and disinfection, commercial feed, and using filtered water by carbon filter, and sealing feed and water. The main routes of swine infection by *T. gondii* include the consumption of meat containing tissue cysts (kitchen garbage), ingestion of oocyst-contaminated water or food, and contact mechanical carriers [5, 14]. These biosecurity practices against ASF also cut off the route of *T. gondii* transmission in swine, especially the oocyst route of transmission. This finding coincided with that of Gazzonis et al. [10]: they also found that the application stricter biosecurity procedures may decrease the risk of *T. gondii* infection on intensive swine farms.

There was no significant difference in the seroprevalence of *T. gondii* infection in pregnancy status, delivery number in sows, and sex. These findings are consistent with other studies [5, 8, 20]. In this experiment, the seropositivity increased with the age of the swine, and the seroprevalence of *T. gondii* was the highest in fattening pigs and the lowest in suckling piglets. Significant differences were found between older and younger swine (*p* < 0.05). This result confirmed that most *T. gondii* infection cases occur after birth, which is consistent with other reports [5, 8]. Here, the seroprevalence of *T. gondii* in adult swine is lower than that of fattening swine. Pregnant sows infected with *T. gondii* may miscarry or give birth to stillborn offspring, and severe toxoplasmosis can lead to death [8, 12]. Some sows may be culled due to decreased production efficiency after infection with *T. gondii.*

Anti-*T. gondii* maternal antibodies could be transmitted through milk to the piglets and begin to decline and disappear after weaning (3–4 months) [8, 9]. Low maternal antibodies in piglets indicated a low seroprevalence of *T. gondii* in sows. In this study, the seroprevalence rates of *T. gondii* in suckling piglets and nursery pigs were 0% and 0.44%, respectively. The low serum-positive rate of *T. gondii* in these piglets also indicated that the risk of *T. gondii* infection in the sows was decreased.

The global seroprevalence of *T. gondii* in swine was summarized by Dubey et al., it was 30.0% (21510/7182) in 2009–2017, and it was 11.2% (98/876) after 2018 [8]. This study showed that the seroprevalence of *T. gondii* antibodies in swine in central China was 1.42%, which was significantly lower than before the ASF outbreak. It indicates that strict biosecurity measures have greatly reduced the risk of *T. gondii* infection in swine from China. However, *T. gondii* still exists in swine and cannot be ignored. It is necessary to cook pork thoroughly, or freeze it completely, before consumption [13].

## Declarations

### Availability of data and material

The datasets used and/or analyzed during the current study are available from the corresponding author on reasonable request.

### Competing interests

The authors declare that they have no competing interests. None of the authors of this report have financial or personal relationships with other people or organizations that could inappropriately influence its content.

### Funding

This study was financed by the Higher Education Teaching Reform Research and Practice Project of Henan Province in 2019 (2019SJGLX006Y), China Agriculture Research System (CARS-35), the Key research projects of Henan higher education institutions (21A230009), and Natural Science Foundation of Henan Province (202300410214).

### Authors’ contributions

WTX collected samples. SLX performed the data analysis and wrote the manuscript. NJ helped in collecting and testing samples. YRY designed the experiment and wrote the manuscript. GPZ, LXZ, and XRL helped in revision of the manuscript. All authors have read and approved the final version of the manuscript.
